# Surgical nodal management in hypopharyngeal and laryngeal cancer

**DOI:** 10.1007/s00405-020-05838-7

**Published:** 2020-02-11

**Authors:** M. C. Ketterer, L. A. Lemus Moraga, U. Beitinger, J. Pfeiffer, A. Knopf, C. Becker

**Affiliations:** Department of Otorhinolaryngology–Head and Neck Surgery, University Medical Centre Freiburg, Killianstrasse 5, 79106 Freiburg, Germany

**Keywords:** Hypopharyngeal carcinoma, Laryngeal carcinoma, Overall survival, cN0, Elective neck dissection, Head and neck cancer

## Abstract

**Objective:**

The aim of this study is to compare pre-therapeutic staging of the loco-regional lymphatic basin and subsequent surgical management in cN0 versus cN+ hypopharyngeal and laryngeal cancer patients.

**Methods:**

We analyzed all hypopharyngeal and laryngeal carcinoma patients treated surgically at a single quaternary medical care and cancer center between 2004 and 2014. We established two groups for patients who underwent neck dissection comparing patients with a low LNR (lymph node ratio) to one with a high LNR. Regarding the cN0 cohort, elective neck dissection was evaluated as a secondary predictor variable. Comorbidities, such as anemia and renal insufficiency, were analyzed as potentially influencing disease-free (DFS) and overall survival (OS).

**Results:**

A total of 310 patients (185 glottic and 125 supraglottic/hypopharyngeal carcinoma) were included. Pre-therapeutic neck MRI-/CT-scan and concomitant neck ultrasound revealed cN+ status in 144 patients resulting in a significant over-staging in 63 patients (44%) who were rated as being pN0 after histological examination. 166 patients were staged cN0 and 21 underwent elective neck dissection (11 local advanced glottic and 10 supraglottic/hypopharyngeal carcinoma). Two cN0 patients showed occult cervical lymph node metastases (10%). Furthermore, we could detect a significant negative impact of the LNR divided by the number of dissected lymph nodes and OS.

**Conclusion:**

The pre-therapeutic clinical evaluation of lymphatic outgrowth is over-staged. OS decreases with increasing LNR divided by the number of dissected lymph nodes. Renal insufficiency and anemia are significant negative factors, decreasing both OS and DFS.

## Introduction

Cervical lymph node metastases are one of the most important factors regarding survival in HNSCC (head and neck squamous cell carcinoma) [12]. Despite modern radiological and clinical diagnostic tools, occult lymph node metastases are still reported [12, 16, 33]. The incidence of elective neck dissection in patients with clinically negative lymph node status depends on the risk of regional metastasis according to localization, stage and size of the primary tumor [13, 17, 31, 32]. Supraglottic and subglottic carcinomas are reported to metastasize more often lymphatically than glottic carcinomas due to scant lymphatic drainage of the vocal folds [12].

The treatment recommendation for clinically N0 staged necks is still controversial. Different authors recommended different therapy options mostly depending on local T-status, histopathological features such as lymphovascular invasion or grading and surgical possibilities for complete resection of the primaries: elective neck dissection (ND) with the surgical resection of the carcinoma itself versus elective neck irradiation versus watchful waiting [12, 23, 29, 31]. Backes et al. [3] described insufficient surgical margins as one of the main risks for reduced overall and recurrence-free survival of laryngeal cancer patients. Böttcher et al. [7] retrospectively analyzed 58 locally advanced staged laryngeal cancers following laryngectomy (LE). They described the importance of midline involvement in preoperative CT scans and a 100% risk reduction without midline involvement for contralateral lymph node metastases. They considered contralateral elective ND as avoidable in locally advanced laryngeal carcinomas without midline involvement [7]. Oztürkcan et al. [28] described a higher risk for contralateral metastases in patients with ipsilateral lymph node invasion with and without extracapsular spread. Furthermore, different authors described the significant reduction of overall survival (OS) in patients with positive lymph nodes [7, 9]. The advantage of ND compared to neck irradiation is the subsequent histopathological examination of occult metastasis to initiate adjuvant therapy [16, 17].

The National Comprehensive Cancer Network [26] recommends pretracheal and ipsilateral ND in total laryngectomized patients for cT3 cN0 glottic and subglottic carcinomas and thyroidectomy with ipsilateral ND and uni- or bi-lateral ND with pretracheal and ipsilateral paratracheal lymph node dissection for cT4a cN0. However, even the current guidelines recommend ipsi- or bi-lateral ND without concretizing indication for elective ND. A lack of studies and clear recommendations lead to progredient subjective elective ND indication [7]. Marks et al. [24] reported 4% lymph node metastases in transglottic and 7–26% in supraglottic carcinomas. Kowalski et al. [22] described a higher risk for occult lymph node metastases in supraglottic cN0 than in glottic cN0 laryngeal cancer patients, but without structured indication for or against elective ipsi- or bilateral ND.

The aims of this study were (1) to determine the influence of surgical lymph node management and lymph node ratio on OS and disease-free survival (DFS) in patients with hypopharyngeal and laryngeal cancer and (2) to examine the incidence of occult cervical lymph node metastases. Furthermore, we aimed to investigate the influence of selected comorbidities such as initial anemia and renal insufficiency on OS following surgical treatment.

## Methods

### Study, design and data collection methods

**Fig. 1:**
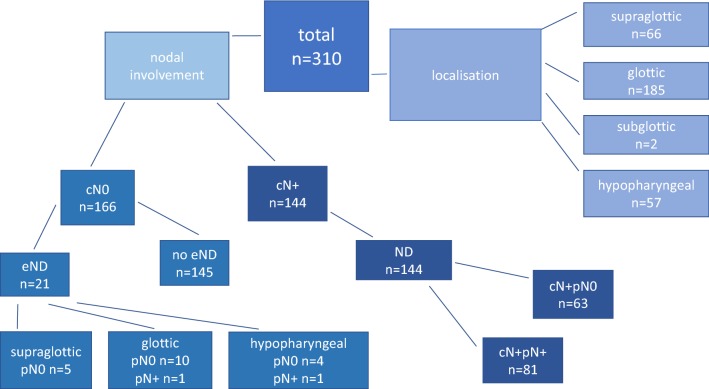
Study cohort

We performed a retrospective analysis of all patients older than 18 years diagnosed with hypopharyngeal or laryngeal squamous cell carcinoma (SCC) and surgically treated between 2004 and 2014 at the University Medical Center Freiburg. We included only patients who underwent surgical therapy (see Fig. 1). We excluded patients who received preoperative irradiation, primary radiochemotherapy, primary radiotherapy and who were under the age of 18 or had been treated at a hospital other than the University Hospital Freiburg. Patients with carcinoma in situ or other histological subtypes were excluded, too.

Histological samples were reviewed by at least two experienced pathologists. We obtained demographic variables such as age and sex, blood levels for anemia and renal function, tumor characteristics (size, stage, histopathology, treatment) and follow-up data retrospectively from patients’ charts. Additionally, we matched our data with the German public cancer register. Panendoscopy had been performed in all patients for histopathological confirmation, defining T-status and subsequent surgical procedure, and evaluation of further malignancies. The preoperative staging included ultrasound of the neck, chest imaging (mainly computed tomography) of the neck and chest and abdominal sonography in all patients. Tumors were staged regarding the UICC (American Joint Committee on Cancer) staging system (7th version).

### Variables

We defined the primary predictor variable as the clinical preoperative nodal status and the surgical treatment. Furthermore, preoperative and pre-existing comorbidities such as anemia and renal insufficiency were defined as secondary predictor variables. We identified and compared patients undergoing elective ND and defined cervical occult lymph node metastases as dissected lymph nodes that were tumor positive without having preoperative suspicion and without fulfilling the metastasis criteria for malignancy published by Ahuja et al. [1]. The primary outcome variable in this study is the OS. The secondary outcome variable is the DFS. Variables of interest (sex, age, year of treatment, stage) were examined and defined as potentially influential on the outcome variables.

### Statistical procedure

We used SPSS (IBM Corp. Released 2015. IBM SPSS Statistics for Windows, Version 24.0, Armonk, NY: IBM Corp.) for statistical analysis. Results were calculated descriptively and mean, standard deviation, maximum and minimum are given in tables and in the text. Comparisons between data were computed using the Wilcoxon test and the level of significance was set at 5.0%. The comparison between different carcinoma locations was calculated with the Levene-test to define homo- versus hetero-geneous variance and the *T* test to define statistically significant differences. Bivariate correlations were computed using the Pearson test. We used the Kaplan–Meier method to analyze survival rates and the log-rank test to calculate differences between OS and DFS.

### Ethics committee

This study was approved by the Hospital’s Ethics Committee according to the Declaration of Helsinki (Washington, 2002).

## Results

### Study and subjects

We included 310 patients diagnosed with hypopharyngeal or laryngeal HNSCC and surgically treated between 2004 and 2014 at the University Medical Center Freiburg. The mean age was 62.2 ± 0.59 years (minimum: 28 years, maximum: 91 years). 157 patients were ≤ 62 years (OS 120.5 ± 5.6 months), 153 patients were > 62 years (OS 88.98 ± 5.1 months). Between those two groups, patients ≤ 62 years showed significantly increased OS (*p* < 0.0001). We included 44 female and 266 male patients but could not find a significant sex-specific difference in mean OS between female (105.4 ± 9 months) and male (103.9 ± 4.3 months) patients (*p* = 0.562). 19 patients had a synchronous second head and neck carcinoma (6.1%) at first presentation after panendoscopy and staging. Two patients (0.6%) were staged with distant metastases (M1) and were excluded from further studies.

### Impact of tumor size (T), grading (G), resection margins (R) and rate of recurrence

**Fig. 2:**
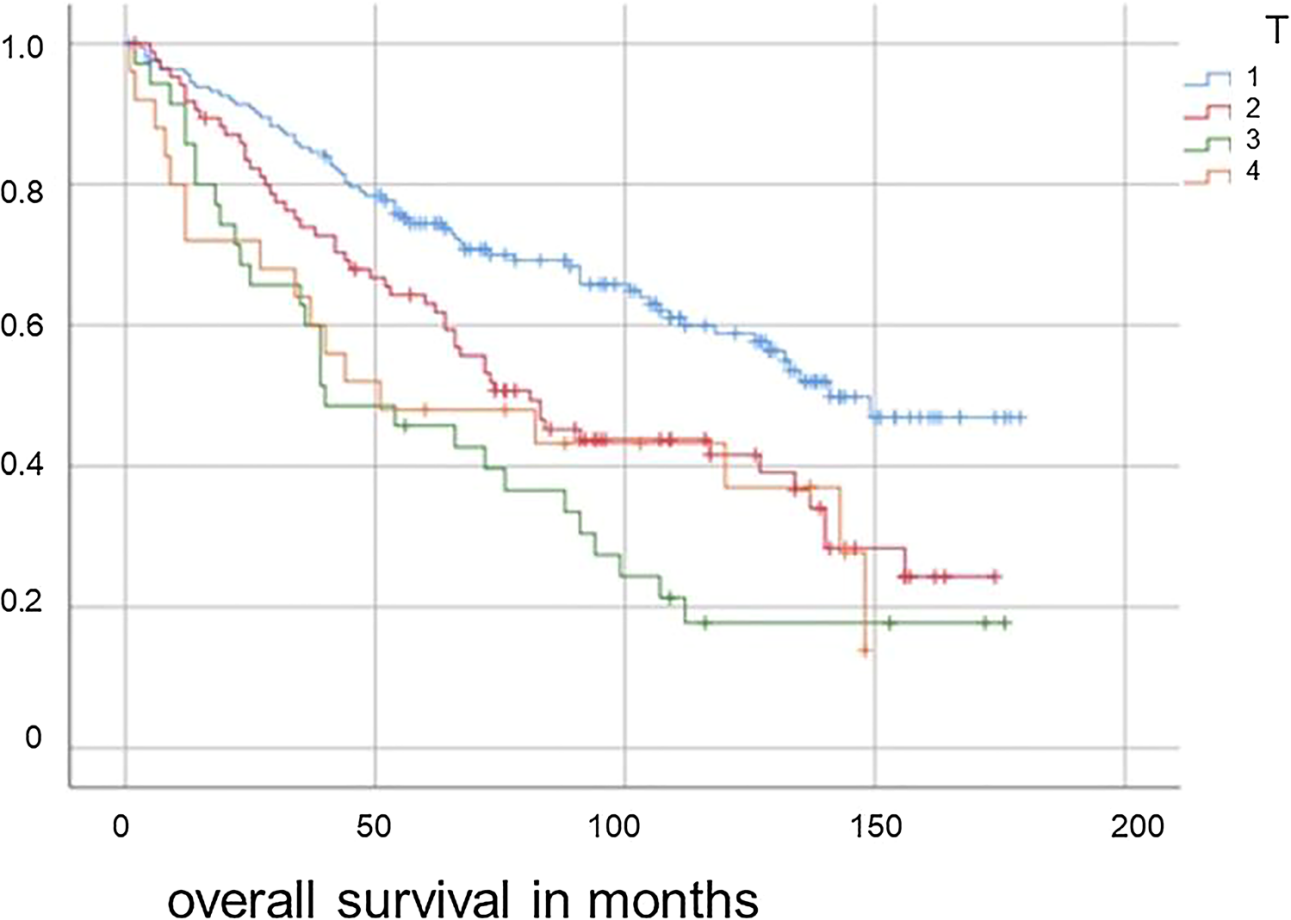
Overall survival for glottis cN0 laryngeal cancer patients regarding initial T- status (*p* < 0.0001)

Most patients had been (oder were) staged cT1 (*n* = 164) before therapy (see Table 1). We could find a significant influence of tumor size on patients with glottic cN0 laryngeal cancer on OS (*p* < 0.0001) and DFS (*p* = 0.006), (Fig. 2) and could detect a significance between T1 and T2–4 (*p* = 0.002) but not between T2 and T3/4. Regarding this, T1 is the tumor size with better OS and DFS rates in cN0 glottic laryngeal cancer. Regarding the cohort of cT2–4 glottic laryngeal cancer, supraglottic, subglottic and hypopharyngeal cancer, we could not detect a significant influence of tumor size on either OS (*p* = 0.936) or DFS.

**Table 1:** Distribution of study group regarding T-, G-, R- and cN-/pN- status and number of patients who underwent ND versus no ND and distribution of pathological nodal status (pN)

	Number of patients	Percentage	Overall survival in months	Standard deviation
pT1	164	52.9	122.5	5.31
pT2	86	27.7	93.79	6.92
pT3	35	11.3	69.45	10.0
pT4	25	8.1	77.0	12.55
G1	21	6.8	105.53	12.8
G2	214	69	107.73	4.7
G3	74	23.9	93.75	7.7
R0	283	91.3	107.66	4.1
R1	15	4.8	77.13	13.54
R2	12	3.9	60.67	19.05
cN0	166	53.5	118.22	5.13
cN1	21	6.8	108.48	14.41
cN2a	5	1.6	61.2	19.5
cN2b	31	10.0	69.62	9.88
cN2c	86	27.7	89.69	7.32
cN3	1	0.3	45.0	3.95
pN0	63	20.3	103.33	8.8
pN1	30	9.7	97.15	12.7
pN2a	3	1.0	64.67	26.6
pN2b	35	11.3	71.75	9.5
pN2c	12	3.9	75.33	19.1
pN3	2	0.6	28.5	16.5
No ND	165	53.2	116.71	5.2

We could not find a significant influence of tumor grading (*p* = 0.246) and most patients showed G2 squamous cell carcinoma (*n* = 214) (see Table 1). However, the resection margins showed significant influence. Initial R0 significantly increases OS compared to R1 and R2 (*p* = 0.018), see Table 1. Furthermore, R0 significantly increases the secondary outcome variable DFS (*p* = 0.002).

Out of 310 included patients, 58 (18.7%) had local or locoregional recurrence of their tumor. Patients with recurrence showed a significantly decreased OS (*p* < 0.0001). Only 2.9% were lost to follow-up (9 patients). 2 recurrences were located supraglottic, 29 in the glottis, 2 subglottic, 10 hypopharyngeal and 14 in other or more than one region.

### Impact of nodal status and elective neck dissection

166 patients were staged cN0 before surgical treatment via ultrasound of the neck (see Fig. 1). 145 patients did not undergo elective ND. Only 21 patients underwent elective ND, of whom 15 patients were diagnosed with supraglottic carcinoma, 4 hypopharyngeal and 2 with subglottic carcinoma. In all cases, elective ND was performed due to tumor localization (hypopharyngeal, subglottic or supraglottic) and and tumor size was additionally decisive in nine patients (> cT3).

Regarding the cN0 cohort (glottic T1cN0 carcinomas excluded), the *T* status showed no significant influence on OS (*p* > 0.05). Furthermore, cN0 was associated with a significant improvement of OS (*p* = 0.003) compared to patients with clinical nodal involvement. Only two of the 21 cN0 patients who underwent elective ND had tumor positive lymph nodes and showed occult cervical lymph node metastases. One patient had a recurrence of a supraglottic laryngeal cancer and underwent laryngectomy with radical ND on both sides. The other one presented with a synchronous hypopharyngeal and epiglottideal carcinoma, both cT1 at first panendoscopy, and underwent surgical treatment of the pT1 carcinoma.

144 patients underwent selective ND. There was no cT1cN0 glottic laryngeal cancer patient in this cohort. A substantial proportion of patients (*n* = 63, 44%) who underwent ND did not have lymph node metastases and were clinically over-staged (*n* = 63). Patients without nodal involvement (pN0) showed significant longer OS but no influence on DFS (*p* = 0.001 and *p* = 0.081). We regarded the ratio of the numbers of metastatic lymph nodes to those of the dissected lymph nodes (lymph node ratio/LNR). Depending on the LNR, 62 patients showed no pathological lymph node (LNR = 0) (OS of 104.61 ± 8.8 months). Defining the LNR as published by Safi et al. [30], we established two groups; one with a low LNR (cut off ≤ 0.006) (in total: 82 patients, OS of 108.05 ± 7.7 months) versus one with a high LNR (cut off > 0.006) (62 patients, OS of 71.86 ± 7.9 months). We found a significant difference regarding the OS (*p* = 0.002) and the DFS between those groups. Interestingly, even if pN0 is not included in the low LNR group, we could detect a significance compared to patients with high LNR (*p* = 0.026). Table 2 shows the LNR divided for different groups, divided by the number of dissected lymph nodes. We could detect a significant correlation between high LNR and overall/disease-free survival in the groups of more than 16 dissected lymph nodes (see Table 2). The group of 26–30 dissected lymph nodes consisted of only 12 patients. Therefore, the not significant correlation is unattended.

**Table 2:** Lymph node ratio ≤ and > 0.06 divided by the number of dissected lymph nodes

Dissected lymph nodes	LNR	Overall survival	*p* value
0–10	≤ 0.06	83.7 ± 22.0	0.065
	> 0.06	66.7 ± 14.8	
11–15	≤ 0.06	106.5 ± 20.3	0.125
	> 0.06	66.2 ± 14.5	
16–20	≤ 0.06	105.5 ± 17.9	0.035*
	> 0.06	56.8 ± 17.7	
21–25	≤ 0.06	143.6 ± 15.7	0.013*
	> 0.06	83.3 ± 15.4	
26–30	≤ 0.06	91.7 ± 20.3	0.068
	> 0.06	70.2 ± 20.4	
> 31	≤ 0.06	105.7 ± 9.9	0.011*
	> 0.06	54.9 ± 17.4	

*Statistical significance of < 0.05

### Comorbidities

**Fig. 3:**
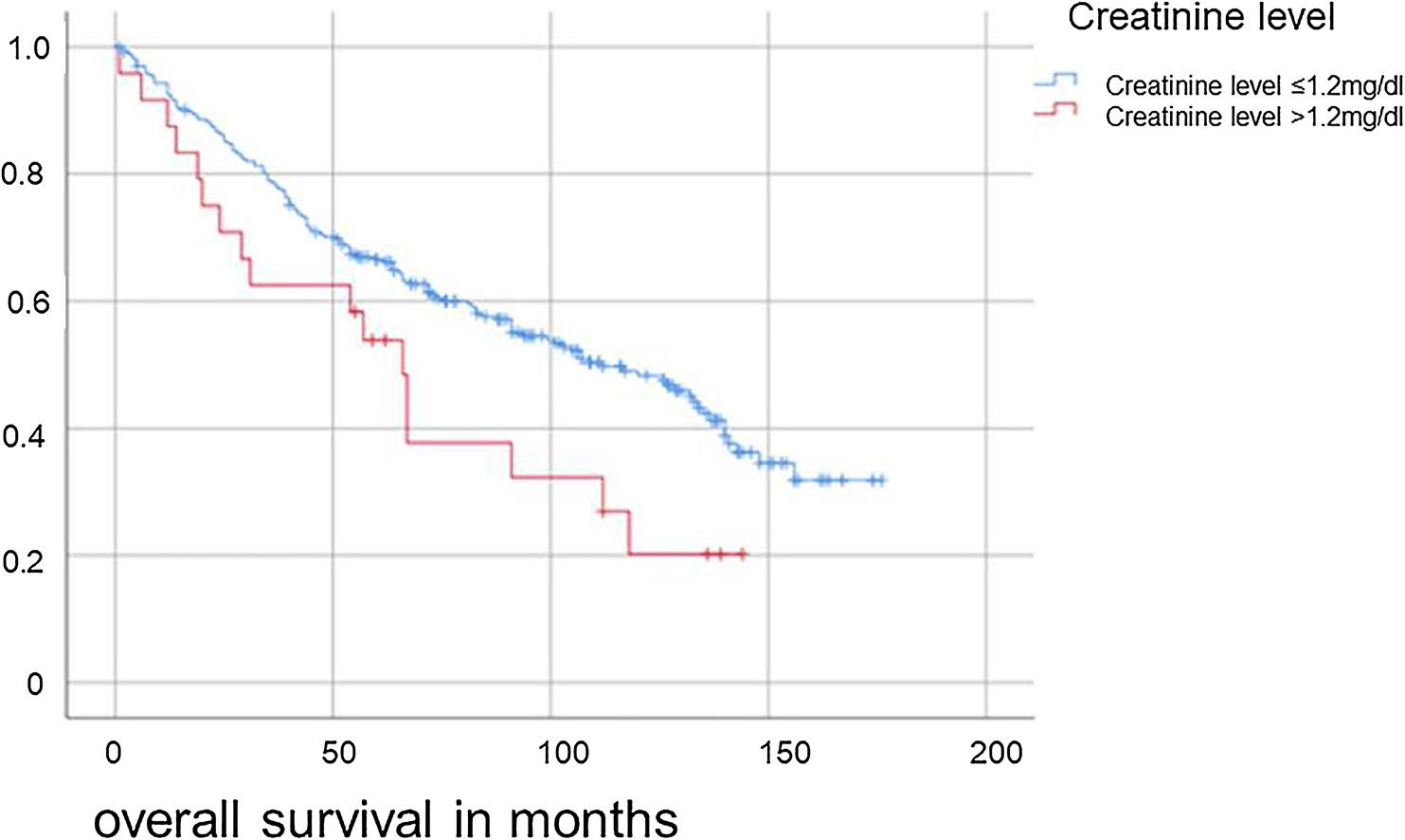
Overall survival for hypopharyngeal and laryngeal cancer patients regarding initial creatinine level (*p* = 0.03)

**Fig. 4:**
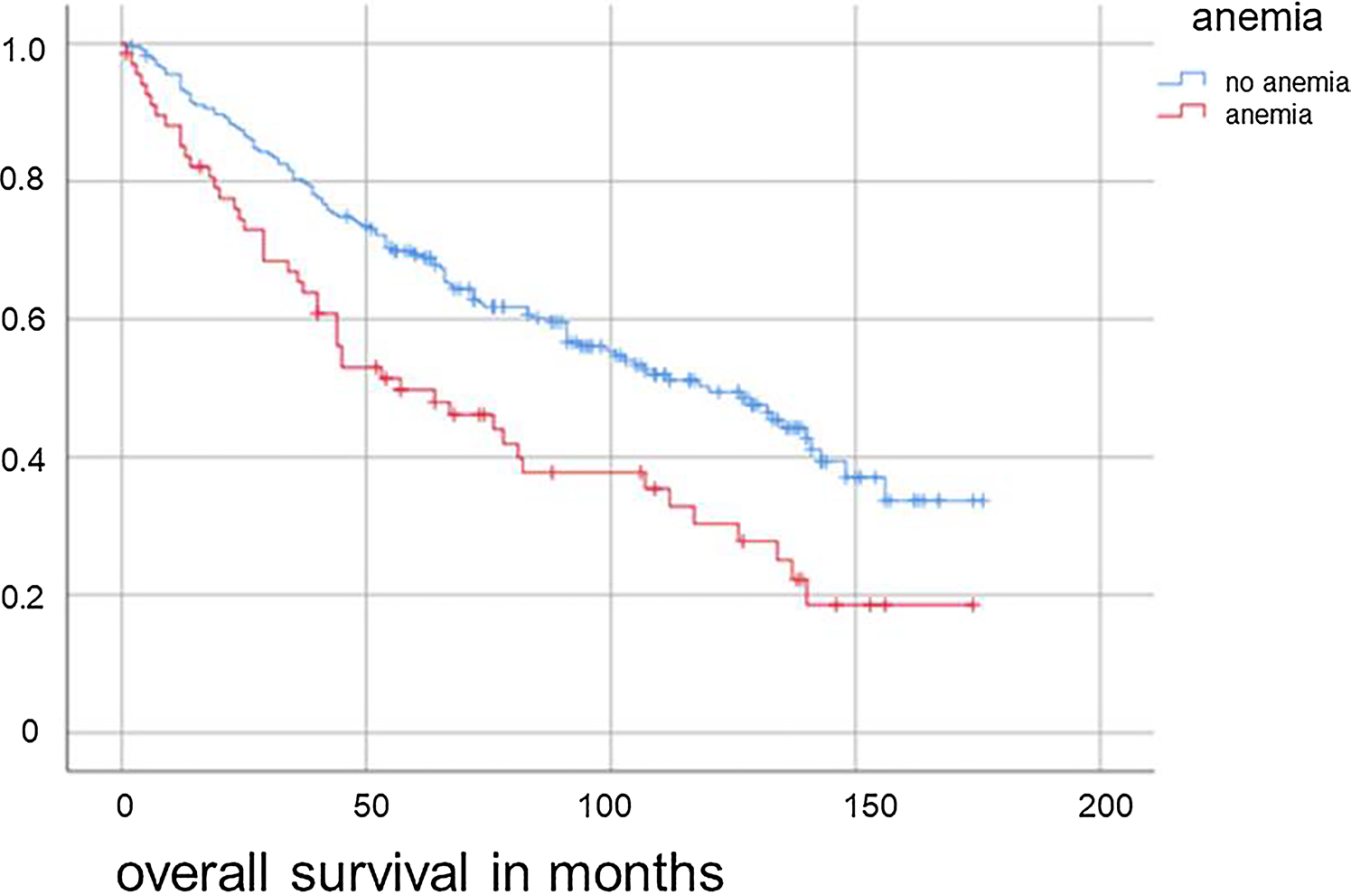
Overall survival for hypopharyngeal and laryngeal cancer patients regarding initial anemia status (*p* = 0.01)

Renal function was evaluated with the creatinine clearance. The mean creatinine was 1.199 ± 0.3 (min: 0.49, max: 3.31). Dividing the patients into two groups, we defined one group with a creatinine level of ≤ 1.2 mg/dl (*n* = 265 patients) and an OS of 104 ± 4.2 months. The second group had a creatinine level > 1.2 mg/dl (*n* = 24 patients) and an OS of 70 ± 10.52 months. We detected a significant difference between these groups (*p* = 0.03) regarding OS and DFS (see Fig. 3). We defined anemia as hemoglobin < 13.5 g/dl or hematocrit of < 41.0% in men and hemoglobin < 12.0 g/dl or hematocrit of < 36.0% in women as published by Williams 2006. The level of erythrocytes was additionally examined. The mean was 4.69 ± 0.029 (min: 3.13, max: 6.78). The mean hematocrit was 42.74 ± 0.23 (min: 30, max: 51.8) and the mean hemoglobin 14.49 ± 0.084 (min: 9.8, max: 17.8). In the total of 293 patients with a preoperative complete lab, 68 showed at least one anemia criterion in hematocrit or hemoglobin. The mean OS of patients with anemia (78.2 months ± 7.9) was significantly lower than of patients without any signs of anemia (108.0 months ± 4.5; *p* = 0.01) (Fig. 4). We could not find any statistical difference between male and female anemic patients. In conclusion, we found a significant negative influence of anemia regarding our primary outcome variable OS (*p* = 0.001) and our secondary outcome variable DFS (*p* = 0.002).

## Discussion

### Impact and importance of nodal status and elective neck dissection

Excluding the glottic T1cN0 carcinomas, the *T* status showed no significant influence on OS (*p* > 0.05). cN0 was correlated with a significant improvement in OS (*p* = 0.003) compared to patients with clinical nodal involvement. Out of 166 cN0 patients included in this study, only 21 underwent elective ND. We could not detect a significantly increased OS or DFS for these supra-/sub-glottic or hypopharyngeal carcinoma patients (OS: *p* = 0.868) (DFS: *p* = 0.547). Only two of these 21 cN0 patients showed occult cervical lymph node metastases, one patient with salvage surgery due to tumor recurrence and the other with synchronous hypopharyngeal and epiglottideal carcinoma. Nevertheless, more than 20% of the cN0 study cohort (glottic cT1N0 excluded) that did not undergo elective ND were treated with adjuvant neck irradiation. A lot of the patients included in this study underwent radiation in another treatment facility and, therefore, we cannot examine this cohort precisely regarding whether they underwent neck irradiation or not. Erdag et al. [12] compared 24 cT2N0 glottic carcinoma patients and found no occult tumor positive lymph node in any of them. They reported an average of 32 dissected lymph nodes (between 8 and 65 lymph nodes per ND). Furthermore, they described no regional failure during a follow-up of two years and concluded that elective ND should not be included in the surgical standard management of cT2N0 glottic carcinoma patients [12]. The results of our study confirm previous studies [12], supporting at least critical indication of elective ND in both hypopharyngeal and laryngeal cN0 cancer patients. In cases of recurrence, salvage ND is easier and with lower complication rates in patients without previous elective ND and/or irradiation [27]. Knopf et al. [21] reported that patients with recurrent disease or functional deficits demonstrated a significant decrease in survival combined with an increase of severe complications. Nevertheless, 63 cN + patients (43.7%) included in this study who underwent ND did not have lymph node metastases and were clinically over-staged. Celakovsky et al. [9] described a discordance of clinical and pathological TNM staging in 32% of 124 included laryngeal cancer patients. They described the nodal status as one of the significant negative influences on OS and DFS. Furthermore, they could show that a disparity in *N*-stage was more frequent in supraglottic laryngeal cancer patients and an overestimated lymph node status, but without influence on the OS or DFS, whereas a disparity in *T*-stage was more frequent in glottic cancer patients with impact significantly decreasing OS and DFS. Furthermore, results for detected positive lymph node metastases in HNSCC cN0 patients vary from 2 up to 58% in the previous literature [9, 10, 15, 18, 25]. This leads to the conclusion that the detection of occult lymph node metastases is dependent on the histopathological detection method, the pathologist and surely also the surgical profoundness. To extrapolate those biases, the LNR divided in different groups depending on the number of dissected lymph nodes, represents an additional prognostic factor.

### Impact and importance of the LNR

In this study, the LNR was divided as published by Safi et al. [30]. We established two groups; one with a low LNR (≤ 0.006) (82 patients) versus one with a high LNR (> 0.006) (62 patients) and found a significant difference regarding the OS and DFS between those groups. Furthermore, we defined different groups for the LNR, divided by the count of dissected lymph nodes (see Table 2). We could detect a significant correlation between high LNR and OS and DFS in all groups. Jacobi et al. [20] defined the LNR as higher and lower than 10% and described it as a relevant prognostic factor in *p* 16 positive, HPV related oropharyngeal cancer patients and an additional prognostic parameter. Our results confirm the prognostic relevance of the ratio between tumor positive lymph nodes and the number of dissected lymph nodes in total also for laryngeal cancer patients. But while previous studies [11, 20, 30] only regarded the LNR in total with the lack of limitation regarding the surgical abilities and variety to dissect from 2 up to 60 and more lymph nodes, we defined groups of dissected lymph nodes and regarded the LNR and its influence on OS and DFS separately. Regarding the LNR described before for other HNSCC, not only is the ratio between positive and dissected lymph nodes important, but also the number of dissected lymph nodes in total. To minimize the dependence on surgical skills to dissect as many lymph nodes as indicated and as possible, we divided our patients in groups ranked from 0 to 10 and to > 31 dissected lymph nodes (see Table 2). In all these groups, we could find a significant influence of the LNR on OS and DFS. Nevertheless, not only the surgical abilities but also the thoroughness of the pathologists influences the LNR. Although preoperative staging methods like ultrasound, CT and MRI have become more and more precise and effective, microscopic examination following ND still remains the gold standard for the detection of lymph node metastases. Nevertheless, if only micrometastases are present in the dissected lymph node, typically localized in the subcapsular sinuses of lymph nodes, pathologists can miss the tumor positivity [8, 9, 14]. Further studies should include this, e.g. by double checking the LNR in the histopathologic samples. Nevertheless, previous studies described up to 30% of false-negative cases in preoperative clinical cN0 staged patients, due to still-lacking accuracy in staging and imaging techniques [2, 4, 9].

### Impact and importance of comorbidities

In this study, we could find a significant influence of the initial renal function and of anemia at first clinical preoperative presentation. We evaluated renal function with the creatinine clearance and divided our patients in two groups with creatinine levels of ≤ and > 1.2 mg/dl and could detect a significant difference between these groups (*p* = 0.03) regarding OS and DFS. Different studies described earlier the significant influence of renal function on OS in oropharyngeal carcinoma [19] but to the best of our knowledge, this is the first study showing significant influence in laryngeal cancer patients in such a large cohort. Nevertheless, further investigations on the impact of systemic comorbidities like renal insufficiency need to be performed on a larger study cohort. Furthermore, the mean OS and DFS of patients with anemia were significantly lower than of patients without any signs of anemia in both male and female anemic patients. Whereas Becker et al. [6] described anemia as having a significant impact on OS in sinonasal cancer, Baumeister et al. [5] described the influence of anemia on survival rates in oropharyngeal cancer. There are case reports and studies with much smaller cohorts, showing a significant coherence between anemia and survival in laryngeal cancer patients, but to the best of our knowledge, this is the largest study focusing on laryngeal cancer and anemia.

## Conclusion

Preoperative, clinical evaluation of lymphatic outgrowth is over-staged and should be performed more precisely due to the discordance found between cN + and pN0 in 44% of the cN0 patients included in this study. The indication for elective ND should be considered precisely, especially in glottic laryngeal cancer patients. Comorbidities such as renal insufficiency and anemia are significant factors decreasing both OS and DFS.

## Abbreviations

CT: Computed tomography
DFS: Disease-free survival
DM: Distant metastasis
HNSCC: Head and neck squamous cell carcinoma
LNR: Lymph node ratio
MRI: Magnet resonance imaging
ND: Neck dissection
OS: Overall survival
